# Homeodomain Interacting Protein Kinase 2 Activation Compromises Endothelial Cell Response to Laminar Flow: Protective Role of p21^waf1,cip1,sdi1^

**DOI:** 10.1371/journal.pone.0006603

**Published:** 2009-08-11

**Authors:** Stefania Mattiussi, Chiara Lazzari, Silvia Truffa, Annalisa Antonini, Silvia Soddu, Maurizio C. Capogrossi, Carlo Gaetano

**Affiliations:** 1 Laboratorio di Patologia Vascolare, Istituto Dermopatico dell' Immacolata, Roma, Italy; 2 Laboratorio di Oncogenesi Molecolare, Dipartimento di Oncologia Sperimentale, Istituto Regina Elena, Roma, Italy; Emory University, United States of America

## Abstract

**Background:**

In the cardiovascular system, laminar shear stress (SS) is one of the most important source of endothelial protecting signals. Physical and chemical agents, however, including ionising radiations and anticancer drugs, may injure endothelial cells determining an increase in oxidative stress and genotoxic damage. Whether the SS protective function remains intact in the presence of strong oxidants or DNA damage is currently unclear.

**Methods and Results:**

To investigate this aspect a series of experiments were performed in which HUVEC were exposed to sub-lethal doses of the radio-mimetic compound Bleomycin (Bleo; 10 µg/ml) which generated free radicals (ROS) without significantly compromising cell survival. Remarkably, the application of a SS of 12 dyne/cm^2^ did not protect endothelial cells but markedly accelerated apoptosis compared to controls kept in static culture and in the presence of Bleo. Experiments with the inducible nitric oxide synthase (iNOS) inhibitor GW274150 significantly reduced the SS-dependent apoptosis indicating that the production of NO was relevant for this effect. At molecular level, the ataxia-telangectasia-mutated (ATM) kinase, the homeodomain-interacting protein kinase-2 (HIPK2) and p53 were found activated along a pro-apoptotic signalling pathway while p21^waf1,cip1,sdi1^ was prevented from its protective action. RNA interference experiments revealed that HIPK2 and p53 were both important for this process, however, only the forced expression p21^waf1,cip1,sdi1^ fully restored the SS-dependent pro-survival function.

**Conclusions:**

This study provides the first evidence that, in the presence of genotoxic damage, laminar flow contributes to endothelial toxicity and death and identifies molecular targets potentially relevant in endothelial dysfunction and cardiovascular disease pathogenesis.

## Introduction

Endothelial cells are physiologically exposed to blood flow which exerts pro-survival and anti-proliferative effects aimed at maintaining endothelial and vascular homestasis [1], [2]. This is achieved with the contribution of multiple molecular effectors including integrins and other adhesion molecules, growth factors, growth factor receptors, paxillin, PI3K/AKT, nitric oxide synthases (NOS), antiapoptotic BCL_2_ family members and components of the cell cycle machinery [1], [2], [3], [4]. In normal conditions, in fact, shear stress (SS), the tangential component of the mechanical forces generated by blood flow, activates p53 along a growth control pathway and up-regulates p21^waf1,cip1,sdi1^ which is important for the SS-dependent pro-survival function[3], [5], [6]. As part of its beneficial properties SS also up-regulates nitric oxide (NO) which contributes to the reduction of damaging reactive oxygen species (ROS) and protects cells from a variety of pro-apoptotic stimuli including those provided by hydrogen peroxide, oxidized LDL and chemically-induced hypoxia[3], [7], [8], [9], [10]. In pathological situations, with elevated intracellular ROS production, NO may turn toward a deadly path resulting in endothelial damage via formation of nitrogen reactive species (RNS) including the highly damaging peroxynitrites [10], [11]. As part of their mechanism of action, therapeutics used as anticancer, such as Bleomycin (Bleo), Adriamycin or ionising radiations, generate elevated levels of intracellular ROS[12], [13] causing genotoxicity and endothelial dysfunction[14], [15]. Specifically, Bleo, which belongs to a family of radio-mimetic glycopeptide antibiotics with potent anti-tumour activities causes important damages to the lung inducing fibrosis and endothelial toxicity[14]. Its cytotoxic and mutagenic effects are thought to be related to the ability of introducing both single- and double-stranded DNA breaks and require the presence of specific cofactors such as a transition metal, oxygen and a one-electron reductant important for the production of elevated intracellular levels of ROS[12], [16].

The mammalians response to DNA damage comprises a highly co-ordinated, yet complex network of proteins that have been categorized as sensors, signal transducers, mediators and effectors of damage and repair. The presence of DNA damage is, in fact, the stimulus to activate a signalling cascade controlled in part by the ataxia-telangectasia mutated (ATM) kinase which phosphorylates the histone isoform 2Ax (γH2Ax) and stabilizes the homeodomain-interacting protein kinase (HIPK2) determining p53 phosphorylation and, according to the extension of damage [17], its activation along a repair/survival or a pro-apoptotic pathway[18]. In this context the role of SS is presently uncertain. Prior reports indicate that SS protects endothelial cells from oxidative stress[8], [19] It is otherwise well known that some of the molecules involved in the SS-dependent signalling are in common with those controlling DNA damage and repair, including p53 and p21^waf1,cip1,sdi1^. Specifically, following DNA damage, p21^waf1,cip1,sdi1^ induction is important for the cell cycle arrest, a mandatory step to properly activate the DNA repair machinery. The absence or unscheduled degradation of this molecule, in fact, leads to accumulation of DNA mutations and apoptosis[20]. Hence, in stress condition p21^waf1,cip1,sdi1^ regulation and/or availability to the SS-dependent signalling cascade could represent an important event with possible functional consequences on the endothelial cell response to mechanical stimuli.

In an attempt to identify mediators of SS function during the endothelial cell response we investigated whether laminar flow contributed to the survival of endothelial cell or played a detrimental role in the presence of DNA damage. Our findings indicate that in the presence of Bleo or other genotoxic agents (see supplemental data) which determined DNA damage and ATM-HIPK2-p53 activation, the application of laminar flow caused a significant increase in endothelial cell death. This effect, was paralleled by a significant limitation in p21^waf1,cip1,sdi1^ expression which further contributed to endothelial toxicity[3].

In conclusion, this work provides the first evidence that, in the presence of specific molecular cues generated by genotoxic damage, laminar flow loses its protective properties creating additional damaging signals. Further, it determines the crucial and protective role of p21^waf1,cip1,sdi1^ against endothelial lethality.

## Materials and Methods

### Cell Culture

HUVEC, human umbelical vein endothelial cells, (Clonetics) were cultured in endothelial cell basal medium (EBM-2, Clonetics) supplemented with an endothelial cell Bullet Kit (Clonetics). In all the experiments cells were used between passage 4 and 6.

### Western Blot analysis

For protein analyses cell lysates were prepared as previously described [3]. The following antibodies were used to detect the proteins of interest: p21 (Santa Cruz Biotechnologies); p53 (Ab1, Oncogene); AcetylLys382p53 (Cell Signaling); phosphoSer15p53 (Cell Signaling); phosphoSer46p53 (Cell Signaling); caspase-3 (Santa Cruz Biotechnologies); iNOS (BD Transduction Laboratories); Nitro-tyrosine (Calbiochem); Tubulyn (Santa Cruz); BAX (Santa Cruz); ATM (Abcam); rabbit anti-HIPK2 (kindly provided by M.L Schimtz), used according to the manufacturer's instruction. Normalization of protein loading was obtained using red-ponceau (Sigma) staining of the membranes and/or coomassie (Invitrogen) staining of the gels.

### NO donor treatment, Nitric oxide production analysis and inhibition of inducible Nitric Oxide Synthase (iNOS)

The cells kept in static condition were exposed to DETANO (500 µM) (Sigma) alone and/or in combination of Bleo. Nitric oxide production was evaluated by the 4,5-Diaminofluorescein (DAF-2 DA) (Alexis) added to the complete medium according to the manufacturer's instruction and as previously described[4]. Inhibition of iNOS enzyme was obtained by GW274150 (Alexis) (10 µM)

### ROS production

2′,7′-dichloro-fluorescein (DCF, Fluka, 30 µM) was added to cell culture medium at the beginning of each experiment. At the end of each time-point cells were incubated with trypsin, detached, washed and further incubated with 3,3′-dihydroethidium (DHE, Molecular Probes, 30 mM) for 15 min in the dark. The ROS production was analysed by flow cytometry.

### Antioxidant treatment

HUVEC were treated with N-acetyl-L-cysteine (NAC) (Sigma) (10 mM) added to the complete medium 16 h before assay. At the end of the experiment the cells were collected for cell death analysis.

### Cell death analysis

For the evaluation of cell death HUVEC were kept 50–70% confluent in the presence of 10 µg/ml of Bleomycin (Aventis) in complete medium and placed in the incubator at 37°C for 1 to 16 h (ST, static condition) or exposed to a laminar SS of 12 dyne/cm^2^, for the same time. Cells were collected at the end of each experiment and the evaluation of the sub-G_1_ DNA content was performed after incorporation of propidium iodide by flow cytometry. Cell death was analysed through Cell Death Detection ELISA (Roche). About 1×10^5^ cells for each condition were processed according to manufacture's instructions.

### RNA interference

For generation of stably interfered endothelial cells Phoenix packaging cells were transfected with p53^−/−^ and HIPK2^−/−^ constructs using Fugene (Roche) to produce retroviral particles as described[4]. The following primers were employed:

Upper HIPK2 5′-AGGAAGAGTAAGCAGCACCAG-3′

Lower HIPK2 5′ -TGCTGATGGTGATGACACTGA-3′

Upper GAPDH 5′ -TCCCTGAGCTGAACGGGAAG-3′

Lower GAPDH 5′-GGAGGAGTGGGTGTCGCTGT-3′

### RNA extraction and Real-time PCR

Total RNA was extracted with Trizol™ (Invitrogen Corp., Carlsbad, CA, USA), reverse transcribed using the High Capacity cDNA Reverse Transcription Kit (Applied Biosystems) according to the manufacturer's instructions and tested by Real-time PCR with the SYBER Green DNA Master Mix (Applied Biosystems) and Applied Biosystems 7500 SDS software. Infected endothelial cells were selected by puromycin. In an independent series of experiments, transient intereference was achieved by using HIPK2-specific (HIPK2i) and universal negative control (UNC) siRNA. Specifically, HIPK2i stealth RNAi sequence (a mix of 3 different sequences) and stealth RNAi negative, Medium GC Duplex, respectively (Invitrogen Corp., Carlsbad, CA, USA). cells were trasduced using Lipofectamine RNAiMAX reagent (Invitrogen Corp., Carlsbad, CA, USA) according to the manifacturer's instructions.

### Adenovirus Infection

For transient gene expression, HUVECs were infected with replication-deficient, recombinant, Adenovirus vectors carrying cDNAs expressing human sense (Ad.sp21) p21[21] and GFP RNA (Ad.GFP) as previously described[3].

### Statistical analysis

Variables were analyzed by Student's t test. A value of P≤0.05 was deemed statistically significant.

## Results

### Laminar SS accelerates endothelial cell death in the presence of Bleomycin

**Figure 1:**
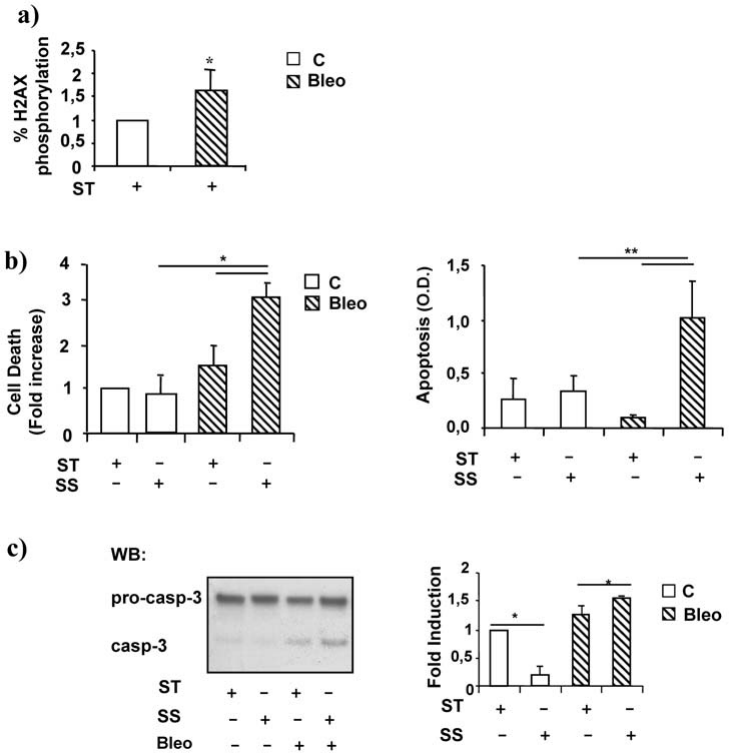
SS induces cell death in endothelial cells exposed to Bleomycin. HUVEC were treated for 16 h with Bleo (10 ug/ml) in static condition ST or exposed to SS. Control cells (open bars); Bleo treated cells (striped bars). a) The graph represents the intracellular content determination of phosphorylated histone 2AX in cell exposed to Bleo. Data are expressed as percent of control. * = p<0.05. b) The graph on the left depicts the result in which cell viability was determined by propidium-iodide staining and FACS analysis. Values are expressed as fold increase compared to static control. The right panel shows the result of free nucleosome evaluation performed by ELISA. Values are expressed in optical density (O.D.). Each experiments was performed three times in duplicate. * = p<0.05; ** = p<0.01. c) A representative western blotting analysis (WB) of caspase 3 cleavage is shown on the left. The inactive precursors (pro-casp-3) and the cleaved form (casp-3) are indicated. The experiments were performed in static culture (ST), in the presence or absence of Bleo (B) and or laminar SS (SS). The right panel shows the average densitometric analysis of three independent experiments. Data are expressed in arbitrary units. * = p<0.05.

HUVEC were exposed to a SS of 12 dyne/cm^2^ for 16 hours (h) in the presence or absence of a sub-lethal concentration of Bleo (10 µg/ml). In this experimental condition the endothelial cell treatment with Bleo alone promoted the chromatin incorporation of the phosphorylated isoform of histone H2AX (γH2AX) (Figure 1**a**). Figure 1**b**, left and right panels, shows the results of experiments in which cell death and apoptosis were evaluated by propidium-iodide staining/FACS analysis (left) and free nucleosomes release (right) respectively. Both were found significantly increased in endothelial cells exposed to SS and Bleo. The evidence of a proapoptotic program activation was further supported by the presence of caspase 3 cleavage (Figure 1**c**, left panel) which reached the highest level, two fold above static control and about seven fold above SS alone, (Figure 1**c**, right panel) in the presence of Bleo and SS.

### Bleo and SS increase oxidative stress and induce iNOS-dependent NO synthesis

**Figure 2:**
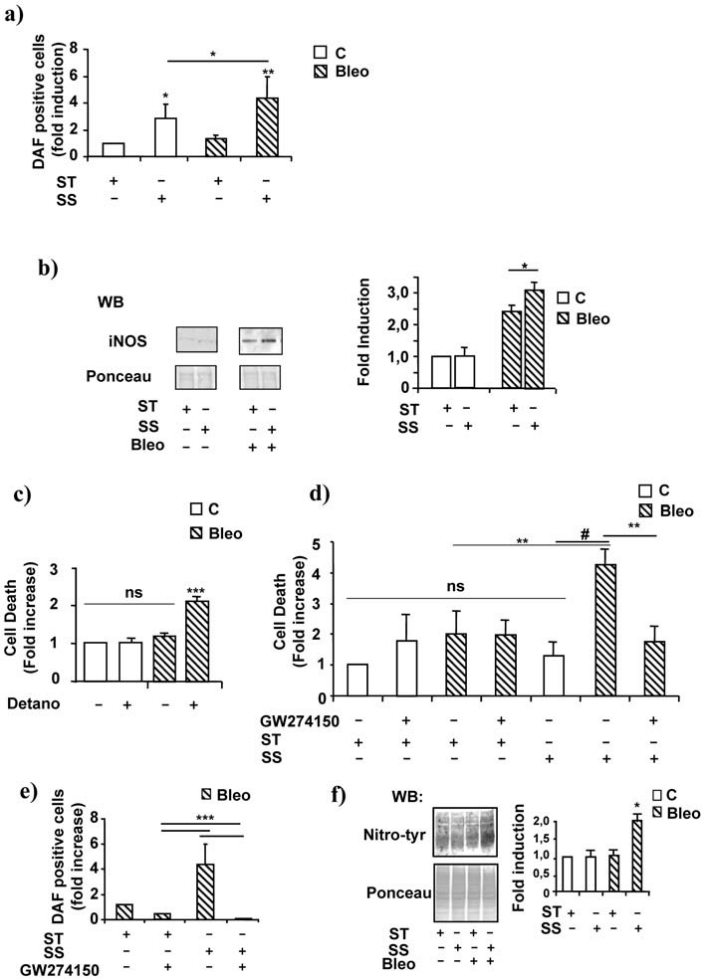
Laminar SS increases iNOS expression and NO production in the presence of Bleomycin. HUVEC were treated for 16 h with Bleo (10 ug/ml) in static condition ST or exposed to SS. Control cells (open bars); Bleo treated cells (striped bars). a) The graph shows the average NO production, in cells cultured in control conditions or exposed to Bleo, SS or both in combination, determined by DAF incorporation and FACS analysis. Data are represented in arbitrary units and are the results of three independent experiments performed in duplicate. * = p<0.05; ** = p<0.01. b) The picture on the left shows a representative western blotting analysis (WB) depicting iNOS protein level in cells kept in static culture (ST) or exposed to laminar SS (SS) in the presence of Bleo (B). Densitometric analysis of three independent experiments expressed in arbitrary units is shown on the right. * = p<0.05. c) The graph on the left shows the effect of the NO donor DETA/NO (500 µM), administered to endothelial cells cultured in static conditions in the presence or absence of Bleo, on cell survival determined by propidium-iodide incorporation and FACS analysis. Data, expressed in arbitrary units, are the results of three independent experiments. Ns = not significant; *** = p<0.001. d) The graph shows the effect of the iNOS inhibitor GW274150 on endothelial cell survival determined by propidium iodide incorporation and FACS analysis. Data are represented in arbitrary units and are the average of three independent experiments performed in static culture (ST) or in the presence of laminar flow (SS). * = p<0.05. ** = p<0.01. # = p<0.005. e) The graph shows the effect of the iNOS inhibitor GW274150 on NO production determined by DAF incorporation and FACS analysis in endothelial cells culture in the presence of Bleo (B). Data are represented in arbitrary units and are the average of three independent experiments performed in static culture (ST) or in the presence of laminar flow (SS). *** = p<0.001. f) The picture shows a representative western blotting analysis (WB) performed by using an anti-nitrotyrosine antibody on total lysates obtained from cells cultured in static condition (ST) or exposed to laminar flow (SS) and/or Bleo (B). The Red Ponceau staining is shown below as loading control (Ponceau). The right panels indicates the average densitometric values expressed as arbitrary units. Data were generated by three independent experiments. * = p<0.05.

As part of its physiological mechanism of action, laminar flow increases the intracellular content of NO by eNOS activation[22]. In our experiments SS increased about 2.5 fold, above basal level, the production of NO in cells kept in normal condition, however, this effect was further enhanced up to 4.5 fold by SS administered in the presence of Bleo (Fig. 2**a**). In patho-physiological contexts, oxidative stress or hypoxia increase the expression of the inducible NOS isoform which synthesizes high levels of NO[23] independently of phosphorylation signals. Figure 2**b**, left and right panels, shows that iNOS expression was induced about 2.5. fold above control by Bleo and even further by the coincident treatment of SS and Bleo. We reasoned that these results could be explained by the Bleo-dependent production of ROS which could be intensified in the presence of SS. Supplemental Figure S1, panels **a** and **b**, shows that Bleo and SS in combination determined a significant increase in the intracellular content of oxygen free radicals evaluated by dichlorofluorescein (DCF) and dihydroethidium (DHE) staining. Notably, DCF, but not DHE, is also able to detect NO[24] which may explain the significant increase in fluorescent signals occurring in the presence of SS and detectable in absence of Bleo as depicted in the supplemental Figure S1, panel **a**.

To evaluate the contribution of NO to the SS-dependent endothelial toxicity occurring in the presence of Bleo an experiment was performed in HUVEC kept in static culture and treated with Bleo in the presence of the NO donor DETA/NO. Figure 2**c**, shows that the NO donor significantly increased cell death indicating that, even in the absence of flow, NO delivered to cells with an elevated intracellular content of ROS contributed to the onset of endothelial toxicity. To investigate the role of iNOS in this process a series of experiments were performed in which the iNOS specific inhibitor GW274150 was added to the culture medium in the presence or absence of Bleo with or without SS. Figure 2**d** shows that GW274150 inhibited the pro-apoptotic response triggered by laminar flow and Bleo. Further, the iNOS inhibitor, virtually abolished the SS-dependent production of NO (Figure 2**e**) suggesting that, in this experimental setting, iNOS may be accounted as the major source of NO.

Although NO is an important positive regulator of endothelial cell function its dual role in apoptosis and survival is well known[11]. In fact, while NO itself is fairly non-toxic and protective, the secondary reactive nitrogen species generated in the presence of free oxygen radicals are potent oxidant and nitrating agents that can modify the structure and function of numerous biomolecules both in vitro, and in vivo[25], [10]. Specifically, in the presence of ROS, the newly synthesized NO easily reacts with them forming highly damaging RNS including peroxynitrites[11]. Figure 2**f**, left and right panels, shows that in the presence of Bleo and laminar SS the total intracellular content of proteins bearing nitrotyrosine residues increased about two fold compared to controls as indicated by western blotting and densitometric analyses. One of the most common consequences of peroxynitrites formation and protein nitration is the functional alteration of important signalling transducers such as the inactivation of PI3K and/or that of other pro-survival molecules[11], [25] followed by the activation of pro-apoptotic mechanisms which may be relevant to the endothelial cell death process described in this manuscript[11].

### p53 activation contributes to the SS-dependent endothelial cell death

Elevated oxidative stress, genotoxic damage and protein nitration represent conditions that turn p53 toward a pro-apoptotic pathway[17]. The presence of DNA damage is, in fact, the stimulus to activate a signalling cascade controlled in part by the ataxia-telangectasia mutated (ATM) kinase which phosphorylates the histone isoform 2Ax (γH2Ax) and stabilizes the homeodomain-interacting protein kinase (HIPK2) determining p53 phosphorylation on serine 15 and 46 and enhancing the expression of proapoptotic molecules, including BCL_2_ family members such as BAX instead of p21^waf1,cip1,sdi1^. Notably, p21^waf1,cip1,sdi1^ is one of the transcriptional targets of p53 which is important for cell cycle arrest and DNA repair. Situations in which p21^waf1,cip1,sdi1^ function results compromised, in fact, often lead to caspase-dependent apoptosis[26].

**Figure 3:**
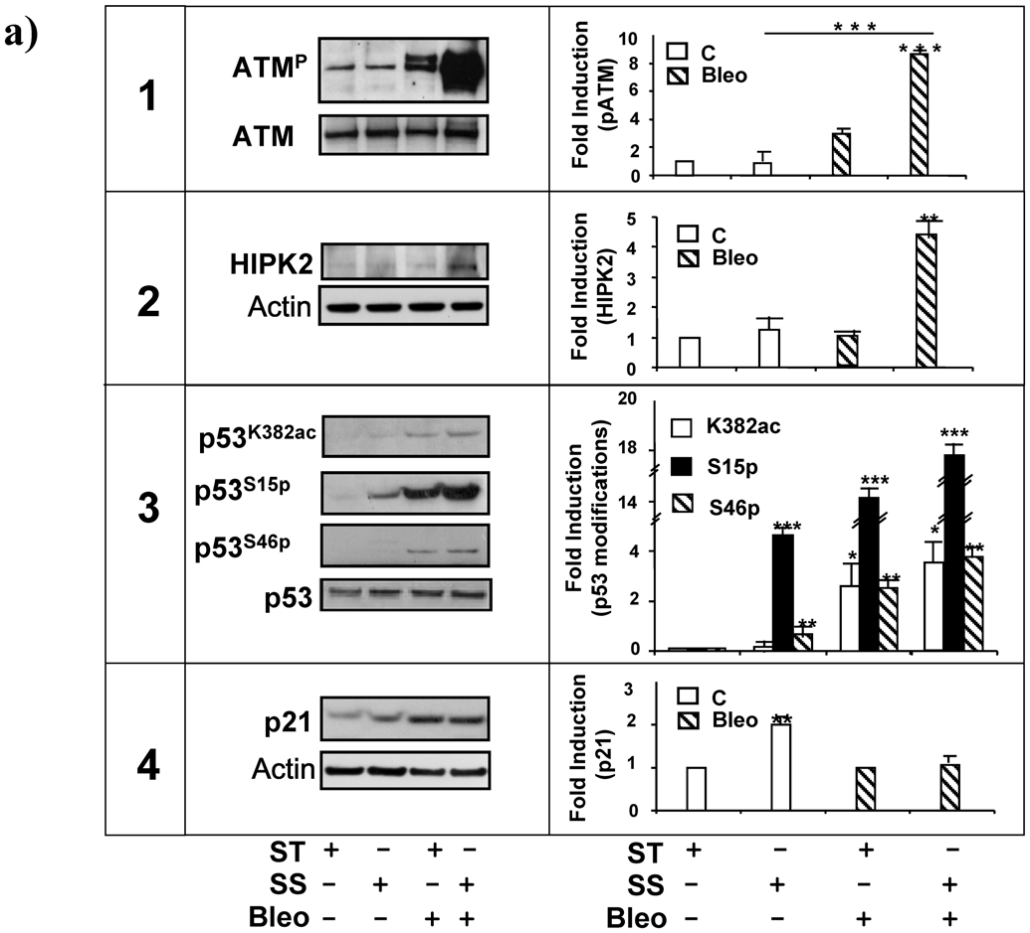
Evaluation of p53 expression and modification in HUVEC exposed to Bleo and SS. HUVEC were treated for 4 h with Bleo (10 ug/ml) in static condition ST or exposed to SS. Control cells (open bars); Bleo treated cells (striped bars). 1. A representative western blotting analysis (WB) of total ATM expression (ATM) and phosphorylation (pATM) is shown on the left. The experiments were performed in static culture (ST), in the presence or absence of Bleo (B) and or laminar SS (SS). The graph on the right depicts the average level of ATM phosphorylation determined by densitometric analysis of three independent experiments. Data are expressed in arbitrary units. *** = p<0.001. 2. The picture on the left is a representative western blotting analysis of HIPK2 expression in cells in cells cultured in static condition (ST), treated with Bleo (B) and/or exposed to a laminar flow (SS). The presence of an unspecific band is indicated by a star on the left. Total actin content is shown as loading control. The graph on the right is the average densitometric analysis of three independent experiments. * = p<0.01. 3. The picture on the left shows the result of a representative western blotting analysis revealing p53 acetylation (p53^K382Ac^) and phosphorylation on Serine 15 (p53^pSer15^) or Serine 46 (p53^pSer46^) in cells cultured in static culture (ST), treated with Bleo (B) and/or exposed to laminar flow (SS). The graph on the right shows the average densitometric values expressed in arbitrary units and obtained by three independent experiments. Data are expressed as percent (%) of control (C). * = p<0.05. ** = p<0.01. *** = p<0.001. 4. The picture (left panel) shows the result of a representative western blotting analysis of p21^waf1,cip1,sdi1^ expression in cells cultured in static condition (ST), treated with Bleo (B) and/or exposed to a laminar flow (SS). The graph on the right is the average densitometric analysis of three independent experiments. ** = p<0.01.

To investigate the effect of Bleo alone or combined to SS in our experimental setting a series of western blotting analyses were performed on ATM, HIPK2, p53 and p21^waf1,cip1,sdi1^. In both conditions, ATM was phosphorylated, however this activator modification was significantly increased in the presence of SS (figure 3**a**, panel 1, left), reaching the highest peak of about 8 fold above static control (figure 3**a**, panel 1, right). Activated ATM kinase stabilizes HIPK2 protein expression[18]. To regulation of HIPK2 in our system was examined in the presence or absence of Bleo, SS or both in combination. Figure 3**a**, panel 2, left, shows that the coincident presence of Bleo and SS determined the highest increase in the intracellular content HIPK2 which raised about four fold above control (Figure 3**a**, panel 2, right). This effect was paralleled by p53 acetylation on Lysine 382 and phosphorylation on Serine 15 and 46[27], as indicated by western blotting and densitometric analyses (Figure 3**a**, panel 3, left and right).

In normal condition, the cell cycle inhibitor p21^waf1,cip1,sdi1^ is elevated by laminar flow and contributes to the SS-dependent growth arrest and endothelial survival [3], [28]. However, figure 3**a**, panel 4, left and right, shows that in the presence of Bleo the SS-dependent increase in p21^waf1,cip1,sdi1^ expression is abrogated.

Altogether, these results suggest that the ATM-HIPK2-p53 signalling pathway activation leads to alteration in the SS-dependent effect on p21^waf1,cip1,sdi1^.

### HIPK2 or p53 knockdown recover endothelial cell survival

**Figure 4:**
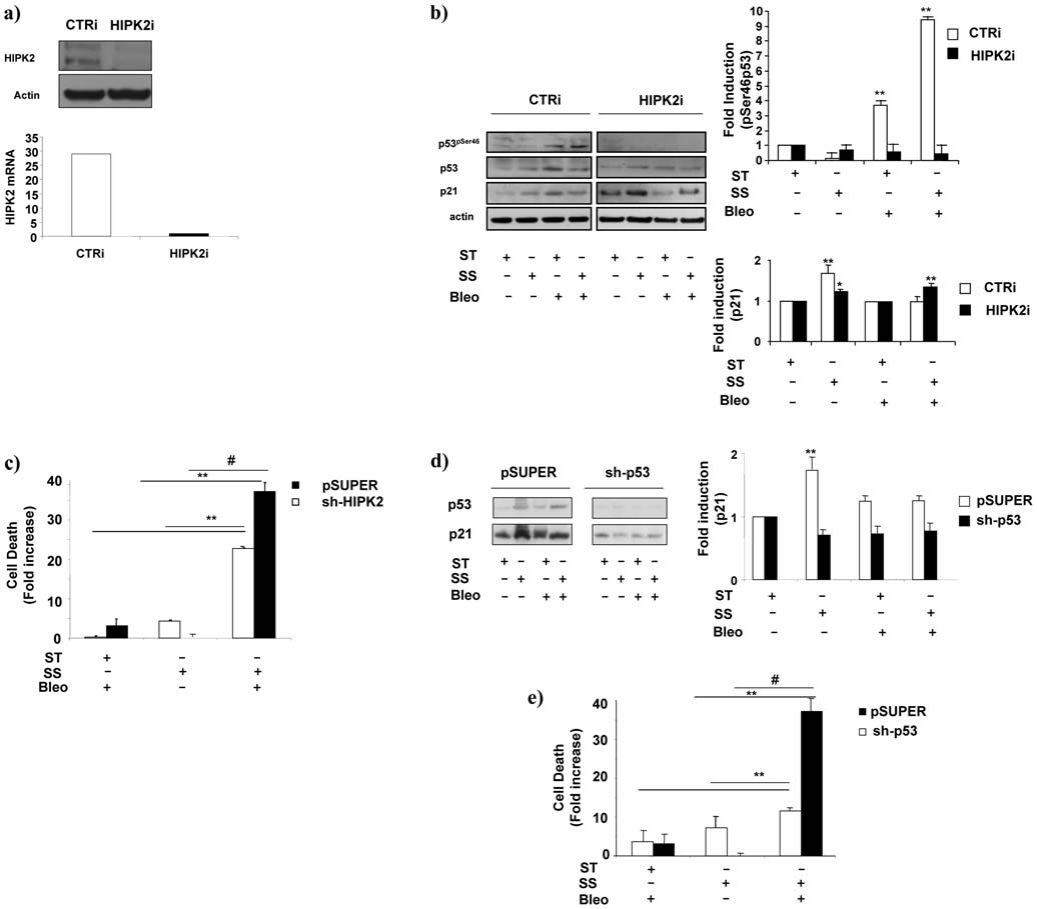
HIPK2 or p53 knock-down influence SS-dependent effects on endothelial cells cultured in the presence of Bleo. HUVEC interfered for HIPK2 or p53 expression were treated for 4 or 16 h with Bleo (10 ug/ml) in static condition ST or exposed to SS. a) The left panel shows a representative western blotting analysis performed in HUVEC transfected with HIPK2 short interfering oligos (HIPK2i) or scrambled controls (CTRi). HIPK2 and actin levels are indicated on the left. The right panel shows the real-time PCR determination of the specific HIPK2 RNA content in CTRi (open bar) or HIPK2i cells (black bars). b) The picture depicts a representative western blotting analysis showing p53 phosphorylation on serine 46 (p53^pSer46^) in cells transfected with scrambled (CTRi) or HIPK2 interfering oligos (HIPK2i). p21^waf1,cip1,sdi1^ expression levels (p21) were evaluated in all the conditions tested. Cells were analysed in static culture (ST), in the presence of laminar flow (SS) with of without Bleo (B). Total p53 and actin content is indicated on the left. The upper and lower graphs on the right show an average densitometric analysis of p53 phosphorylation in serine 46 and p21^waf1,cip1,sdi1^ expression respectively as determined in CTRi (open bars) or in HIPK2i (black bars). Data, obtained from three independent experiments, are expressed in arbitrary units. ** = p<0.01. c) The graphs shows the results of three independent experiments aimed at determining survival in control (pSUPER) or HIPK2 interfered cells (sh-HIPK2). The average data of three independent experiments were generated by propidium iodide staining and FACS analysis after 16 h of static culture (ST), laminar flow exposure (SS) with or without Bleo (B). ** = p<0.01. # = p<0.03. d) The picture shows the expression level of p53 and p21^waf1,cip1,sdi1^ determined by western blotting in cells infected with the control (pSUPER) the p53 (sh-p53) or the HIPK2 (sh-HIPK2) interfering retroviruses. Data were generated from cell cultured on static culture (ST), exposed to laminar flow (SS) with or without Bleo (B). P53 (p53) and p21^waf1,cip1,sdi1^ (p21) bands are indicated on the left. The lower graph depicts the average densitometric analysis of p21^waf1,cip1,sdi1^ levels determined in three independent experiments. pSUPER, open bars, sh-p53 black bars, sh-HIPK2 (striped bars). ** = p<0.01. e) The graphs shows the results of three independent experiments aimed at determining survival in control (pSUPER) or p53 interfered cells (sh-p53). The average data of three independent experiments were generated by propidium iodide staining and FACS analysis after 16 h of static culture (ST), laminar flow exposure (SS) with or without Bleo (B). ** = p<0.01. # = p<0.03.

To functionally investigate this aspect endothelial cells with a reduced content of HIPK2 or p53 were generated by transient or stable RNA interference. Figure 4**a**, left and right panels, shows the efficacy of HIPK2 knock-down (HIPK2i), obtained by specific short interfering RNA oligos, at protein (left) and RNA level (right). Figure 4**b**, left panel shows that in this condition Ser46 phosphorylation on p53 was abolished in spite of the presence of Bleo and/or SS compared to control cells (CTRi) in which p53 Ser46 phosphorylation increased about 9 fold above basal level (Figure 4**b**, right panel). Remarkably, in cells with a reduced content of HIPK2, the effect of SS on p21^waf1,cip1,sdi1^ expression was restored in the presence of Bleo as indicated by western blotting (Figure 4**b**, left) and densitometric analyses (right).

To provide insights about the functional role of HIPK2 and that of p53 in the Bleo and SS-dependent cyto-toxicity a series of experiments were performed in cells in which RNA interference was obtained by retroviruses bearing short-hairpin interfering oligonucleotides directed to HIPK2 (sh-HPK2) or p53 (sh-p53). Figure 4**c**, shows that endothelial cells with a reduced HIPK2 content were significantly resistant, compared to their pSuper control, to the induction of the cell death triggered by laminar SS in the presence of Bleo. As expected, similar results were obtained in cells with a reduced content of p53 (figure 4**d and e**).

These results indicate that the coincident treatment with SS and Bleo realizes a condition in which HIPK2 is activated an negatively influences the SS-dependent effect on p21^waf1,cip1,sdi1^ which is important for the pro-survival response to laminar flow.

### p21^waf1,cip1,sdi1^ rescues laminar flow protection of endothelial cells exposed to Bleo

**Figure 5:**
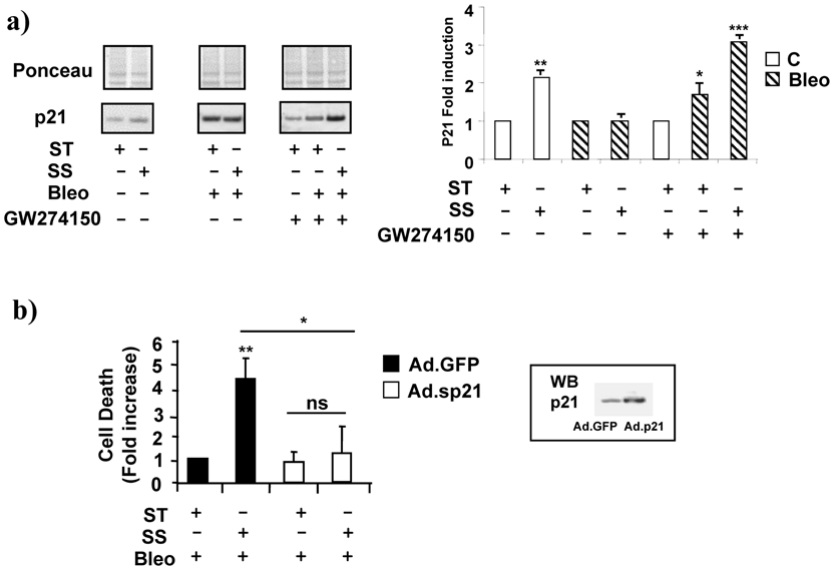
p21^waf1,cip1,sdi1^ is regulated by iNOS and protects cells from SS-dependent cell death in the presence of Bleo. HUVEC were treated for 16 h with Bleo (10 ug/ml) in static condition ST or exposed to SS. Control cells (open bars); Bleo treated cells (striped bars). a) The picture shows a representative western blotting analysis of p21^waf1,cip1,sdi1^ levels in cells cultured in static conditions (ST), in the presence of laminar flow (SS), with or without Bleo (B) and/or the iNOS inhibitor GW274150. The relative p21^waf1,cip1,sdi1^ band is indicated on the left. Red Ponceau is shown as loading control (Ponceau). The graph on the right shows the average value of p21^waf1,cip1,sdi1^ levels in the different experimental conditions. Data were generated from three independent experiments. * = p<0.05. b) The graphs shows the effect of the adenovirus (Ad.p21) mediated p21^waf1,cip1,sdi1^ overexpression (open bars) in cells culture in static condition (ST), in the presence of laminar flow (SS) with or without Bleo (B). A GFP adenovirus was used as control (black bars). The inset on the left shows a western blotting analysis of p21^waf1,cip1,sdi1^ in control (GFP) and Ad.P21 infected cells (p21). * = <p0.05. ** = p<0.01.

Our prior work revealed that the ability of laminar flow to regulate p21^waf1,cip1,sdi1^ expression is important protecting endothelial cells from growth factor deprivation and hypoxia-dependent cell death [3]. Although little information is available about the role of p21^waf1,cip1,sdi1^ in the presence of oxidative stress and genotoxic damage, the experiments depicted in figure 5**a** **,** left and right panels**,** show that, the iNOS inhibitor GW274150 fully restored the SS-dependent regulation of p21^waf1,cip1,sdi1^ suggesting that iNOS, as well as HIPK2, is important to prevent p21^waf1,cip1,sdi1^ induction. Figure 5**b** demonstrates that adenovirus-mediated p21^waf1,cip1,sdi1^ over-expression prevents endothelial cell death in all the conditions tested indicating an appropriate ratio between basal and SS-inducible levels of p21^waf1,cip1,sdi1^ may play an important role in the SS-dependent protective function of endothelial cells.

## Discussion

This manuscript describes the effect of SS that, applied to endothelial cells in the presence of a source of genotoxic damage, determined a significant increase in cell death. This effect was evident in the presence of Bleo which mechanism of action is based on the rapid and prolonged increment of oxidative stress and the consequent production of DNA single- and double- strand breaks. Notably, similar results were obtained with Adriamycin and UV radiations (see figures S2, S3 and S4).

Prior work reports that laminar SS protects endothelial cells against a variety of damaging stimuli including chemical hypoxia, oxidative-LDL, and hydrogen peroxide [4], [9], [10], [20]. Nevertheless, the effect of SS in the presence of genotoxic damage has never been tested. Our findings indicate that, in conditions which generate potent oxidative and mutagenic reactions, SS does not prevent but, on the contrary, contributes to cell death. This phenomenon may either depend on the formation of multiple species of radicals including oxygen free radicals, hydroxyls and peroxynitrites either on the alteration of some of the epigenetic functions of NO[29], [30], [31] which may ultimately lead to a dysregulated gene expression and capacity of DNA repair[32]. During our attempt of molecularly dissect this process the following experimental points were established: 1) laminar flow applied to cells treated with Bleo determined a marked increase of the inducible form of the endothelial nitric oxide synthase; 2) as a consequence the intracellular levels of NO were sharply boosted, a process which contributed to the formation of additional damaging reactive nitrogen species including peroxynitrites; 3) the highly pro-oxidant environment generated by this process activated a proapoptotic signalling cascade characterized by ATM and HIPK2 activation and a series of p53 post-transduction modification compatible with its role as a pro-apoptotic promoter; 4) in the presence of Bleo and SS, the cell cycle inhibitor p21^waf1,cip1,sdi1^ up-regulation was prevented with negative consequences on its antiapoptotic function so important in endothelial cells[3] suggesting that critical circumstances may exist in which the presence of SS provides additional damaging signals leading to endothelial cell death rather then protection. Although the role of iNOS in the genotoxic response to Bleo has been previously described and it is known that reducing or inhibiting its function ameliorates cellular responses to Bleo[33], it is novel that to iNOS or HIPK2 may be ascribed a role in the control of p21^waf1,cip1,sdi1^ expression and that p21^waf1,cip1,sdi1^ alone is sufficient to overcome the toxic effect of Bleo restoring the physiologic function of laminar flow.

**Figure 6:**
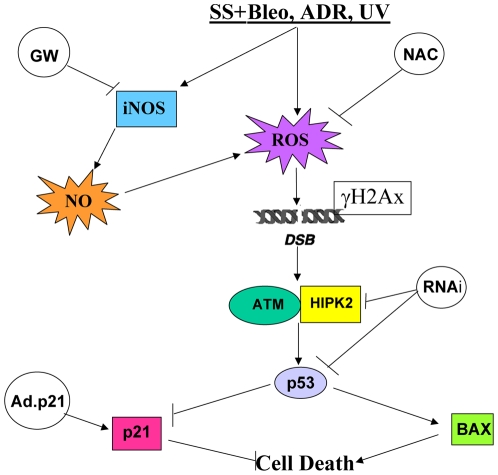
Schematic representation of SS-dependent signalling pathways activated in the presence of genotoxic damage leading to endothelial cell death and preventative interventions. Endothelial cells exposed to laminar shear stress (SS) and various sources of genotoxic damage (Bleo, Doxo, UV) generate reactive oxygen species (ROS) and up-regulate the inducible nitric oxide synthase at protein levels (iNOS) which, elevating the intracellular content of nitric oxide (NO), contributes to the increase of the intracellular ROS content. Genotoxic damage generates single and double DNA strand breaks which determine Ataxia-telangectasia mutated kinase (ATM) activation and phosphorylated histone 2AX incorporation (γH2AX) in DNA repair foci. ATM is known to regulate the homeodomain-interacting protein kinase 2 (HIPK2) which participate to the DNA repair process regulating p53 function by Serine 46 phosphorylation and activation along a DNA repair or proapoptotic signalling pathways. The latter regulated by proapoptotic members of the BCL_2_ protein family (BAX). In this condition, the SS-and p53-dependent cell cycle inhibitor and anti-apoptotic molecule p21^waf1,cip1,sdi1^ (p21) is inhibited contributing to endothelial cell death. Interventions aimed at reducing the ROS level, the production of NO or the intracellular content of HIPK2 and p53 reversed the SS-dependent endothelial cell death. Remarkably, the adenovirus-mediated (Ad.p21) p21^waf1,cip1,sdi1^ over-expression revealed an important protective role of this molecule preventing apoptosis in the presence of SS and genotoxic agents.

Taken altogether these data represent the first experimental evidence that SS properties are strongly influenced by the intracellular environment which determines the endothelial cell response to mechanical stimuli. Consequently, the presence of local flow alterations worsened by co-factors such as metabolic alterations, inflammatory processes or oxidative stress, may hamper the SS-dependent positive regulation of p21^waf1,cip1,sdi1^, determining the reversal of flow pro-survival functions (see schema in Figure 6). Noteworthy, molecular interventions aimed at restoring the appropriate intracellular levels of p21^waf1,cip1,sdi1^ successfully rescued the anti-apoptotic properties of SS. In conclusion p21^waf1,cip1,sdi1^ emerges from this study as an essential keystone molecule at the crossroad between homeostasis and pathogenesis of endothelial cell dysfunction.

## Supporting Information

Figure S1 Laminar SS increases ROS production in endothelial cells exposed to Bleo.
(3.97 MB TIF)

Figure S2 Doxorubicin and UV radiation compromise SS-dependent protection of endothelial cells.
(3.37 MB TIF)

Figure S3 Doxorubicin and UV irradiation prevent the SS-dependent up-regulation of p21waf1,cip1,sdi1.
(0.70 MB TIF)

Figure S4 Forced p21waf1,cip1,sdi1 expression prevents apoptosis in the presence of Doxo but not after UV irradiation.
(80.33 MB TIF)
