# Supplementary figures and images for: Homeodomain Interacting Protein Kinase 2 Activation Compromises Endothelial Cell Response to Laminar Flow: Protective Role of p21waf1,cip1,sdi1
Source: PLoS One. 2009 Aug 11;4(8):e6603. doi: 10.1371/journal.pone.0006603 (PMC2719102; doi:10.1371/journal.pone.0006603)

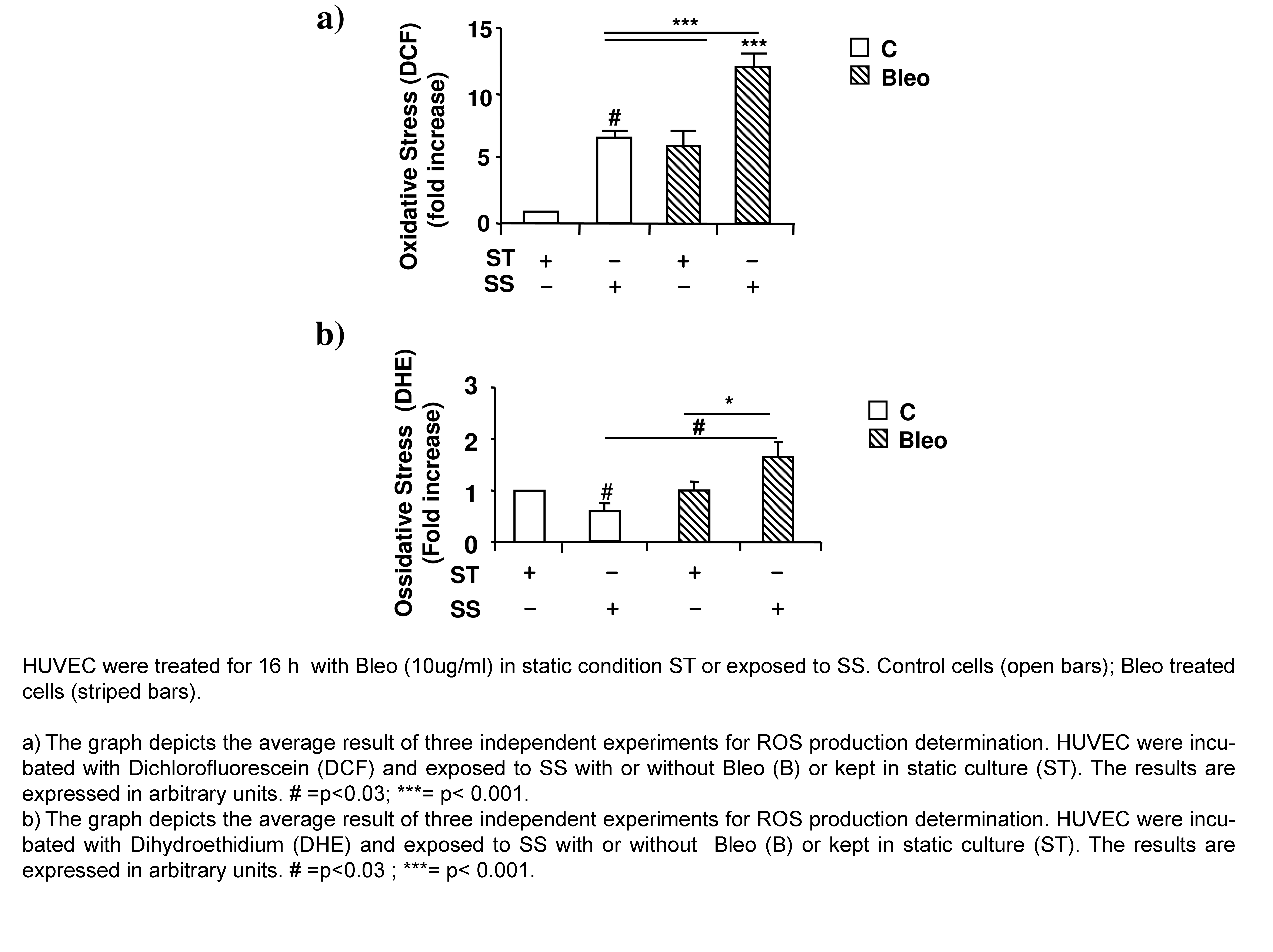

Supplement: Figure S1 — Laminar SS increases ROS production in endothelial cells exposed to Bleo. (3.97 MB TIF) [file pone.0006603.s001.tif]

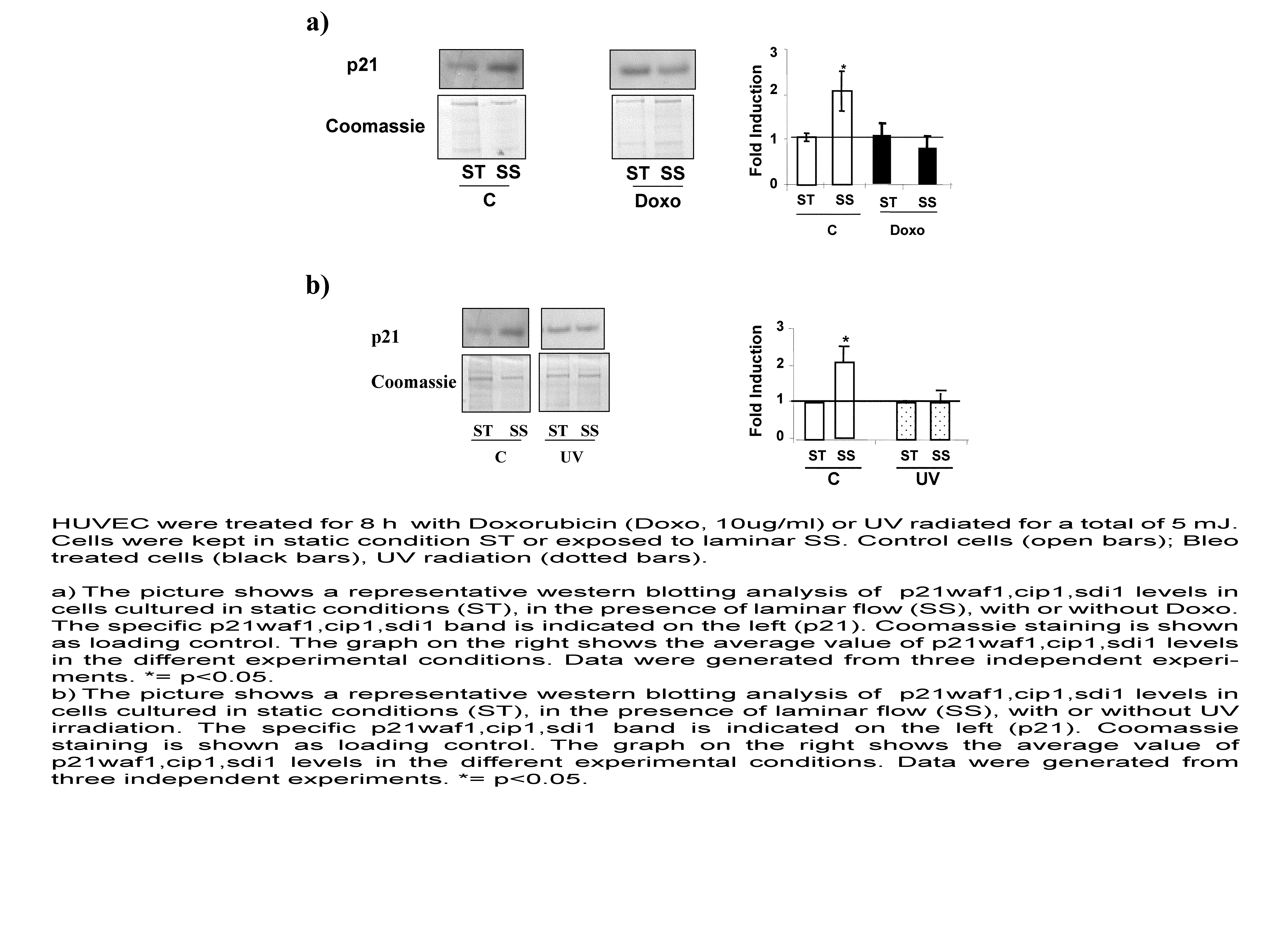

Supplement: Figure S3 — Doxorubicin and UV irradiation prevent the SS-dependent up-regulation of p21waf1,cip1,sdi1. (0.70 MB TIF) [file pone.0006603.s003.tif]
